# The Encoding of Individual Identity in Dolphin Signature Whistles: How Much Information Is Needed?

**DOI:** 10.1371/journal.pone.0077671

**Published:** 2013-10-23

**Authors:** Arik Kershenbaum, Laela S. Sayigh, Vincent M. Janik

**Affiliations:** 1 National Institute for Mathematical and Biological Synthesis, Knoxville, Tennessee, United States of America; 2 Department of Evolutionary and Environmental Biology, University of Haifa, Haifa, Israel; 3 Woods Hole Oceanographic Institution, Woods Hole, Massachusetts, United States of America; 4 School of Biology, University of St Andrews, St Andrews, United Kingdom; University of Rennes 1, France

## Abstract

Bottlenose dolphins (*Tursiops truncatus*) produce many vocalisations, including whistles that are unique to the individual producing them. Such “signature whistles” play a role in individual recognition and maintaining group integrity. Previous work has shown that humans can successfully group the spectrographic representations of signature whistles according to the individual dolphins that produced them. However, attempts at using mathematical algorithms to perform a similar task have been less successful. A greater understanding of the encoding of identity information in signature whistles is important for assessing similarity of whistles and thus social influences on the development of these learned calls. We re-examined 400 signature whistles from 20 individual dolphins used in a previous study, and tested the performance of new mathematical algorithms. We compared the measure used in the original study (correlation matrix of evenly sampled frequency measurements) to one used in several previous studies (similarity matrix of time-warped whistles), and to a new algorithm based on the Parsons code, used in music retrieval databases. The Parsons code records the direction of frequency change at each time step, and is effective at capturing human perception of music. We analysed similarity matrices from each of these three techniques, as well as a random control, by unsupervised clustering using three separate techniques: k-means clustering, hierarchical clustering, and an adaptive resonance theory neural network. For each of the three clustering techniques, a seven-level Parsons algorithm provided better clustering than the correlation and dynamic time warping algorithms, and was closer to the near-perfect visual categorisations of human judges. Thus, the Parsons code captures much of the individual identity information present in signature whistles, and may prove useful in studies requiring quantification of whistle similarity.

## Introduction

The complexity of dolphin vocalisations has long fascinated scientists, and inspired numerous attempts to classify and decode them. Dolphins and other cetaceans produce a wide range of vocalisations, including tonal whistles, clicks, and burst pulses. Several species, including the bottlenose dolphin (*Tursiops truncatus*) produce, among others vocalisations, a call that is highly specific to the individual. These calls have been coined “signature whistles” [1]. Signature whistles are particularly common during capture-release events [2], when an animal is isolated from its group [3], and when groups join [4]. Their presumed function is to aid individual identification and group cohesion, and animals in captivity have been shown to produce both their own signature whistles and copies of those of their pool-mates [5], [6]. Dolphins respond preferentially to the signature whistles of familiar individuals [7], and playback experiments with artificially generated sounds have shown that animals can distinguish between the signature calls of different individuals using only the frequency modulation profile of the tonal elements in the call [13]. This contrasts with the mechanism of individual recognition in many other species, in which individuals use information encoded in acoustic parameters such as call length, scalar measures of fundamental frequency, and harmonic composition; e.g. red deer [8] and rock hyrax [9]. However, although it is known that dolphins use the whistle frequency modulation as a cue in recognition, it is not known what features of the whistle modulation encode individual identity.

Several studies have shown that human observers can reliably identify individual dolphins from a spectrographic representation of their signature whistles [2], [3], [10]. The spectrographic representation is a very different modality (visual) to the original signal (acoustic), yet it is possible that both dolphin acoustic inspection and human visual inspection of the calls make use of the same cues for individual identity. In particular, the spectrogram provides a strong visual representation of the frequency modulation (FM) of the whistle, but does not emphasise amplitude modulation (AM) which is used both in birds [11] and humans [12] to convey information. This implies that dolphin identity is encoded in FM rather than AM (although the possibility of the presence of redundant information in AM cannot be excluded), and this idea was confirmed empirically by Janik et al. [13]. Thus, similar to the situation in bird song research, classification of whistles by eye has been a common method in signature whistle research.

Several studies have tried to find methods that classify whistles by other means. A variety of methods have used computer algorithms to classify whistles in the absence of any information on the underlying categories used by dolphins themselves. Examples are correlation of fixed-point sampling [14] and polynomial fitting [15], [16]. However, it is unclear how the resulting whistle types map onto the whistle categories used by dolphins. In fact, it has been demonstrated that the correlation of fixed-point sampling cannot find signature whistle categories [2], [10]. Much more successful at signature whistle classification have been studies that used dynamic time warping to minimise the total square differences between frequency profiles [17] or used time warping and an adaptive resonance theory neural network for classification [18]. However, both are time consuming and potentially use more information from a whistle than is necessary for successful classification. A method that tries to minimise the information needed to classify signature whistles correctly would be helpful for whistle classification in large data sets and would allow us to develop testable hypotheses regarding how dolphins may perform classification themselves.

A fruitful field to use as a basis for this effort might be human musical recognition and encoding. “Expert” recognition of musical tunes appears to involve a “lossy” representation of the original signal, i.e., one where much of the data has been discarded [19]. A number of these techniques do not preserve the global characteristics of the tune [20]. Prominent among these is the Parsons code [21], which has been extensively used for the retrieval of tunes from music databases [22]. The Parsons code represents a frequency profile as a series of “up”, “down”, and “constant” samples, thus recording only the direction of frequency change. Variations on the Parsons code also indicate the relative magnitude of the frequency change as well as the direction. Nonetheless, the Parsons code has been shown to be effective at capturing the essential information in a tune [22].

If the individual information in dolphin signature whistles is preserved under a Parsons-type encoding technique, then we would expect a good clustering performance of Parsons-encoded whistles, since most machine-learning algorithms can be expected to benefit when the input data are pre-processed to include only relevant features [23]. This would in turn imply that these, or similar features, contain sufficient information to allow dolphins to identify whistles, whether or not the Parsons-features are actually used by the animals for decoding. Either way, it would allow us to develop more effective algorithms for assessing whistle similarity, by focussing on those elements that are sufficient for the task.

## Methods

We reanalysed the same data described in Sayigh et al [2], which consisted of 400 signature whistles: 20 whistles each from 20 identified individuals. The whistles were recorded in Sarasota Bay, Florida, using suction-cup hydrophones during brief capture-release events for health assessments described in [24]. The 20 dolphins were selected randomly from a library of over 150 individuals with at least 200 whistles for each individual, and 20 of these whistles were selected randomly for each animal. Technical details on the recording equipment and digitisation can be found in Sayigh et al [2]. We used a discrete Fourier transform of length 256, with a Hamming window of 1 ms, and 50% overlap to create spectrograms.

**Figure 1:**
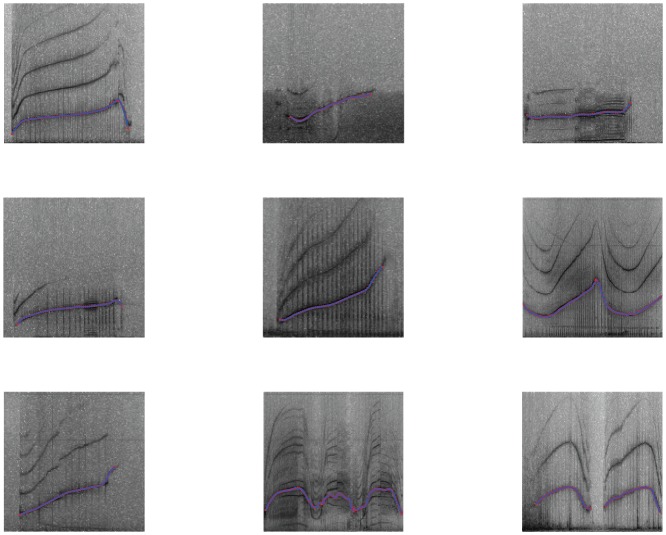
Examples of the spline-smoothed whistles (blue line), on top of manually extracted curves (red points).

We also reused the visual clustering from 10 inexperienced human observers (i.e. unfamiliar with data set), as described in the same study. Each observer was asked to group spectrograms of all 400 whistles into classes by frequency profile similarity, without having any information indicating how many individual dolphins were represented, how many whistles there were for each dolphin, or what guidelines should be used for grouping similar whistles. For the automatic clustering, we extracted the whistle frequency profiles obtained by sketching the course of the dominant frequency manually on the spectrogram, using custom visualisation software to assist manual whistle tracking. This provided a set of time-frequency points of variable length, depending on the duration of the whistle. We then filtered these data using a cubic-spline technique [25], to capture the essential shape of the whistle (Figure 1).

We examined three separate metrics for whistle similarity: (1) the correlation metric (CM) suggested by McCowan & Reiss [14], (2) a simlarity matrix of time-warped whistles (DTW) [17], and (3) a Parsons code-like metric (PC) as described below. We then used three separate clustering algorithms to group together similar whistles: (a) k-means clustering, (b) hierarchical clustering, and (c) an adaptive resonance theory neural network ART [26]. We performed all calculations in Matlab R2012a (Mathworks, Natick, MA).

For each of the metrics, and each of the clustering algorithms, we measured the success of the clustering assignment using the Normalised Mutual Information [27], [28]. Normalised mutual information (NMI) is a single metric that measures how well a clustering scheme matches the true classes (individual dolphin identity); NMI takes values near 1 when clusters are each exclusively composed of a single class, and near zero for random clusters. NMI is defined as:
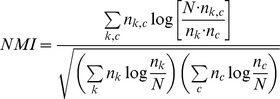
where *n_c_* is the number of whistles from dolphin *c*, *n_k_* is the number of whistles in cluster *k*, *n_k,c_* is the number of whistles from dolphin *c* in cluster *k*, and *N* is the total number of whistles. Note that high values of NMI do not preclude that a particular class be divided into multiple clusters, but higher scores are obtained when the number of clusters is close to the number of true clusters.

For the correlation metric (CM), we followed the technique suggested by McCowan & Reiss [14], and reproduced by Sayigh et al [2], and sampled the time-frequency curve at 60 equally spaced time points, to produce a 400×60 matrix of feature-space vectors. We then performed principal component analysis (PCA) to extract those eigenvectors that best capture the variation in this feature space, selecting those eigenvectors with eigenvalues greater than 1.0, as suggested by McCowan & Reiss [14]. This led us to select the 16 strongest features. Since the hierarchical clustering algorithm requires a proximity matrix rather than a feature-space matrix, we also calculated a 400×400 proximity matrix using the Euclidean distance between pairs of the whistles in the 16-dimensional feature space [29].

**Figure 2:**
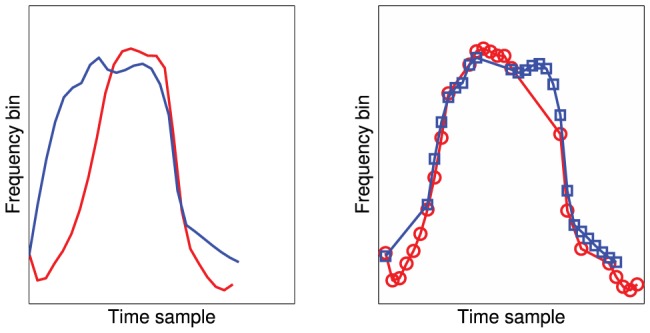
An example of the dynamic time-warping matching of two whistle profiles. The left frame shows the original signals on arbitrary time and frequency axes. The right frame shows the red sample having undergone a dynamic time-warping transformation to produce the minimum least-squares distance from the blue sample. Note how the spacing of the points in the curve have been varied.

The dynamic time-warping (DTW) metric measures the minimum distance between individual whistles, when the x-axis (time) spacing between data points is allowed to vary freely (see Buck & Tyack [17] for a more detailed discussion of the use of DTW for cetacean vocalisations). As well as cetaceans, DTW has been widely used for analysing the vocalisations of birds [30], and other mammals [31]. This technique gives improved matching particularly when salient features in the whistle profile (such as peaks) may occur at slightly different times (Figure 2). We calculated the DTW metric [32] for each pair of whistles, resulting in a 400×400 proximity matrix. Since the k-means and ART clustering algorithms require a feature-space matrix rather than a proximity matrix, we used multidimensional scaling [33] to generate a lower dimensional feature space in which the Euclidean distance between points best corresponded to the proximity matrix generated by DTW. To maintain consistency with the CM technique, we fixed the size of this feature space at 16 dimensions. Note that this treatment of the DTW data is not the same as that used in ARTWARP [18], another software program used in cetacean call classification.

**Figure 3:**
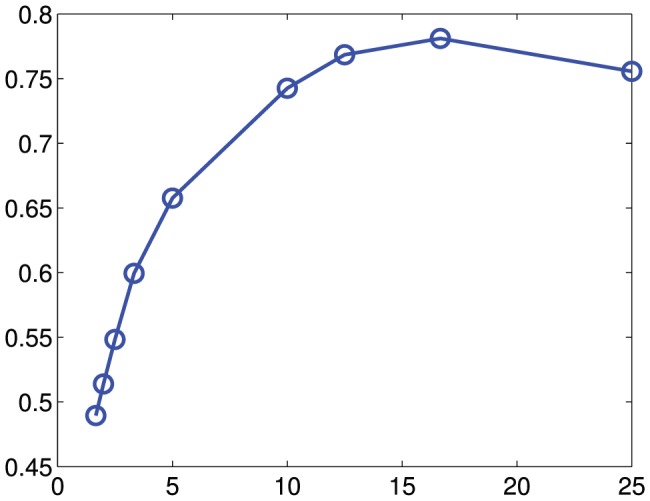
Sensitivity of the algorithm performance (normalised mutual information) as the number of Parsons segments is varied.

To calculate the Parsons code metric, we resampled each whistle into 10 equally spaced segments, and recorded whether the mean frequency of each segment was higher (“up”), lower (“down”), or within a tolerance of one pixel (“constant”) of the previous segment. We chose 10 segments since this provided a compromise between loss of information (few segments) and convergence on the continuous-time analysis (many segments). This produced a nine digit, base-3 code for each whistle. In a preliminary investigation, we verified the choice of a 10 segment code by measuring the clustering success (as measured by the Normalized Mutual Information using the k-means clustering algorithm) when the number of segments is varied between one and 25 (Figure 3). This indicated that a number of segments below 10 or above 20 resulted in decreased performance. We then compared each pair of whistles and measured the edit distance using the Needleman-Wunsch algorithm [34], [35]. Edit distance measures the minimum number of insertions, deletions, and substitutions required to convert one string into another [36], and has been used previously [37] to create a distance metric between syntactic sequences in animal vocalisations. As with the DTW metric, we also generated a 16-dimensional feature-space matrix using multidimensional scaling.

A number of authors [38], [39] have proposed an extension to the Parsons code in which the relative magnitude of the frequency change is recorded, in addition to the direction. *n*-Parsons encoding uses *n* levels for either rising or falling frequency. For instance, a 1-Parsons encoding gives the original “up”, “down”, and “constant” codes, whereas in a 3-Parsons encoding, the frequency change in each segment is assigned to one of three absolute magnitude groups (i.e. large, medium, and small), providing a total of seven classes of frequency change: “large drop”, “medium drop”, “small drop”, “no change”, “small rise”, “medium rise”, “large rise”, and therefore a base-7 encoding. Following Pauws [38] and Müllensiefen & Frieler [39], we defined the groups “large rise” and “large drop” as together containing the 10% largest magnitude changes over the data set as a whole. Larger *n* values provide a more faithful encoding of the original information, but require more storage and processing ability. Therefore, we considered the lowest *n* value that results in an improvement of identity information encoding as the optimum encoding strategy for this technique. We calculated the *n*-Parsons metric where *n* = [1], [8], i.e. base 2*n*+1 encodings, *n* = 1 being the original Parsons code, and selected for further analysis both the optimum *n*-value encoding, and the 1-Parsons encoding. In addition to the CM, DTW, and PC metrics, we also generated a 400×400 random proximity matrix, and its corresponding 400×16 feature-space matrix as a control.

**Figure 4:**
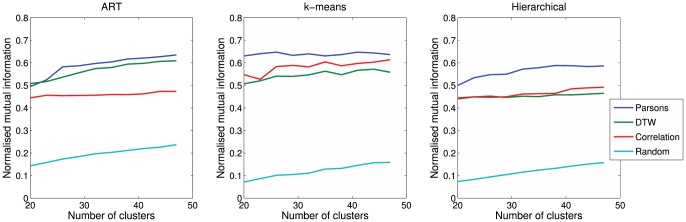
Sensitivity of the algorithm performance (normalised mutual information) for all metrics (Parsons, DTW, correlation, and random control), and all clustering algorithms (ART, k-means, and Hierarchical), as the number of clusters is varied.

We used three separate and very different clustering algorithms to exclude the possibility of our results arising from the idiosyncrasy of a particular clustering algorithm; different clustering algorithms may produce different results when applied to the same data [29]. First, we applied the k-means algorithm (Matlab function *kmeans*), as proposed by McCowan & Reiss [14], and used by Sayigh et al [2]. We chose to cluster the data into 30 groups, 50% more than the number of dolphins present, to allow for some variation in signature whistles within individuals, but without reducing the clustering task to triviality by allowing a very large number of clusters. The results of the k-means algorithm can be strongly affected by the choice of the number of clusters, so we additionally tested the sensitivity of our results using between 5 and 50 clusters for the k-means and other algorithms. This showed some variation in the final results, but the relative performance of the different algorithms remained largely unchanged at reasonable cluster sizes (Figure 4).

For hierarchical clustering, we used the Matlab function *cluster*, which performs agglomerative clustering, using a complete (i.e. longest-distance) linkage map. To retain consistency with the k-means clustering, we restricted the hierarchical tree to 30 clusters.

We also used an unsupervised neural network clustering algorithm based on the Adaptive Resonance Theory (ART) approach [26]. This technique has been used successfully, e.g. by Deecke & Janik [18], who developed software called ARTWARP, which combines DTW and ART to cluster cetacean vocalisations. For our purpose of comparing proximity metrics and clustering algorithms independently, we used separate implementations of DTW [32] and ART [40]. However, note that this study does not include a comparison with the ARTWARP performance since ARTWARP is generally used with contours sampled every 5–10 ms and since we treated the DTW data differently from ARTWARP.

Having generated cluster assignments for all 400 whistles using each of five metrics (CM, DTW, 1-PC, optimum *n*-PC, and the random proximity matrix), and each of the three clustering algorithms (k-means, hierarchical, and ART), as well as the single cluster assignment from the visual observers, we measured the success of the clustering assignment using the Normalised Mutual Information (NMI).

For analysing the human visual clustering taken from Sayigh et al [2], we calculated the standard error of the NMI across each of the observers. For the automatic metrics, we calculated the standard error on a population of 100 NMI measures, generated by bootstrapping the whistle data, each time randomly selecting 80% of the whistles. Each of these populations, and the visual clustering population, were compared using univariate ANOVA with a post-hoc Tukey test, in IBM SPSS v20 (IBM Corp, Armonk, NY).

## Results

**Figure 5:**
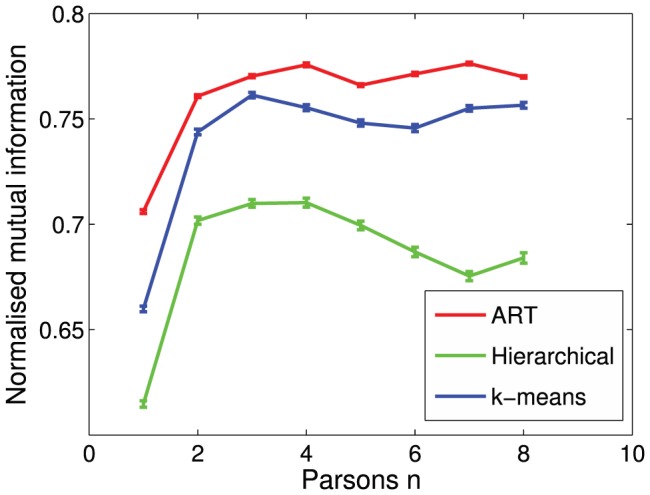
Normalised mutual information for different *n*-Parsons encodings, with each of the clustering algorithms. Error bars indicate the standard error of the 100 bootstrapped iterations.

The success of retrieving identity information from *n*-Parsons encoded whistles rises sharply for *n* = 2, and saturates around *n* = 3 or *n* = 4 for all clustering algorithms (Figure 5). Although the change in NMI from *n* = 1 to *n* = 2 is fairly small (ART: 8%, Hierarchical: 14%, k-means: 12%), it is nonetheless quite marked, and consistent between the different algorithms. We selected *n* = 3 as the optimum Parsons encoding for the remainder of the analysis, as it appears to be the lowest *n* value to maximise success.

**Figure 6:**
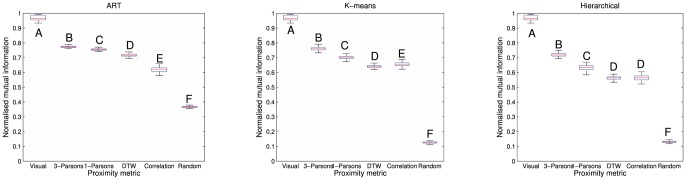
Boxplots showing the normalised mutual information (NMI) for the different encodings, compared to human clustering (A). The panels show ART clustering (left), k-means clustering (middle) and hierarchical clustering (right), using the different proximity metrics. Each box shows the 25^th^ and 75^th^ centiles, with the median indicated as a red line. Whiskers show the extreme values (±2.7σ) using the Matlab *boxplot* function. Letters indicate Tukey HSD post-hoc tests at the *p*<0.05 level, following ANOVA.

Visual clustering produced near-perfect allocation of whistles to individual dolphins, with NMI values between 0.90 and 0.99 (mean 0.96). All of the automatic metrics produced much lower NMI values (Figure 6), with the highest NMI obtained from the 3-Parsons metric using the ART clustering (NMI = 0.774±0.001 SE).

The automatic algorithms produced NMI values between 0.52 and 0.79, and all provided better clustering than the random control matrix. For each of the three clustering algorithms, analysis of variance (ANOVA) showed a significant difference between the encoding techniques (ART: F(5,510) = 20758, *p*<0.001, k-means: F(5,510) = 40718, *p*<0.001, Hierarchical: F(5,510) = 19224, *p*<0.001). A post-hoc Tukey HSD test showed that both Parsons code metrics performed significantly better than the DTW or CM techniques (Figure 6) for each of the clustering algorithms (*p*<0.001 in each case). Similarly, the DTW metric performance was better than CM using the ART clustering, slightly worse than CM using k-means, and no different with hierarchical clustering. The post-hoc test also showed that the 3-Parsons metric gave significantly higher NMI than all the other metrics, including the 1-Parsons metric, for all clustering algorithms, and visual classification was significantly better than all of the automated metrics, for all clustering algorithms (*p*<0.001 in each case).

## Discussion

As noted in previous studies [2], human visual comparison of spectrograms provides an extremely accurate clustering of dolphin whistle spectrograms. Of the three metrics we examined, the 3-Parsons code gave consistently higher scores than the CM or the DTW approaches, although it was still far inferior to human visual classifications, giving results about 25% less accurate. This difference was statistically significant in each of three unrelated clustering algorithms: k-means, hierarchical, and adaptive resonance networks. In contrast, the relative performance of both the CM and DTW metrics varied according to which clustering algorithm was used.

Although we do not propose that dolphins make use of a Parsons-like comparison of whistles to identify individuals, our bottom-up, or model-based, approach to call categorisation [41] indicates where sufficient information is encoded, should the animals take advantage of this mechanism. We attach particular significance to the fact that the Parsons code provided effective clustering with very little information. A nine digit 3-Parsons code can differentiate 7^9^≈2^25^ combinations (25 bits), whereas a 60-point frequency profile on a spectrogram with a frequency resolution of 128 can differentiate 128^60^ = 2^420^ combinations (420 bits). When reduced by principal component analysis (PCA) to 16 dimensions, this falls to 128^16^ = 2^112^, still far more than the information contained in the Parsons code. As Beyer et al [23] showed, clustering with high dimensionality is likely to fail, since the distance between points becomes indistinguishable in high dimensional space. Most machine learning approaches attempt to reduce dimensionality, while retaining discriminating information. The performance of the Parsons code algorithm strongly implies that the information captured by this encoding is sufficient for the differentiation between individuals in this data set. The saturation of the *n*-Parsons performance at *n* = 3 implies that the maximum amount of identity information that can be encoded by a Parsons metric can be captured using the ±3 notation. It is not possible to infer biological significance directly from this; however, if animals were to use such an encoding technique, it would be possible for them to distinguish 75% of identity information in these signature whistles by relying on gross segmentation of the whistle profiles into “large drop”, “medium drop”, “small drop”, “no change”, “small rise”, “medium rise”, and “large rise” segments. Testing this hypothesis would require playback experiments in which signature whistles were modified to vary correlation and dynamic time-warping characteristics, while maintaining Parsons code-like features.

Fripp et al [42] showed that signature whistles are developed by dolphins as calves, and appear to be learned from other members of their community, but with modifications rendering them individually distinctive. The form of this modification, and how differences between individuals are encoded, may indicate how dolphins construct new vocalisations to identify themselves as a new individual. Many animal species produce vocalisations in which individuals can be distinguished using the acoustic features of the call. Koren & Geffen [9] performed discriminant function analysis on a selection of vocal characteristics, such as element length and timing, in the calls of the rock hyrax (*Procavia capensis*) and used this information to differentiate between the calls of known individuals. Charlton et al [43] demonstrated that individual identity is encoded in the amplitude modulation of the calls of the giant panda (*Ailuropoda melanoleuca*). However, both these and most other cases of individual vocal identity appear to rely on voice cues, which arise as a by-product of anatomical and physiological differences between individuals [8], usually without the involvement of vocal learning [44]. In contrast, bottlenose dolphins encode identity information in the learned frequency modulation of the signature whistles [13]. Signature whistles are therefore of particular importance in animal vocal communication research, as they are an example of “designed individual signatures” [44], and “not a by-product of individual anatomical or physiological differences as in most other animals” ([2]),

Both the ability of dolphins to recognize the modulation pattern of the fundamental frequency [13], and the ability of human observers to produce accurate clustering of dolphin signature whistles by visual examination of spectrograms of the fundamental frequency, imply that sufficient identity information is encoded in the frequency modulation pattern. However, the mechanism by which humans cluster whistle spectrograms is unknown. In the acoustic domain, it has been shown [19], [20] that human experts can recognise musical patterns encoded with highly lossy techniques such as the Parsons code, and that these encodings are an efficient way to store the distinguishing features of musical tunes in a compact database [22], from which retrieval is fast and reliable. It would therefore not be surprising if animals make use of similar lossy encodings to distinguish between the calls of individuals, as this would require far fewer cognitive resources. However, our current knowledge on whistle classification methods is equivocal. Ralston & Herman [45] showed that dolphins can learn to categorise signals by absolute parameter values or by the frequency modulation pattern of the signal independent of the frequency band it is in. For signature whistles, Caldwell et al [1] suggested that dolphins can recognise signature whistles even if only exposed to a short part of the frequency profile, while Harley [6] was unable to reproduce this result with a trained dolphin. We hope that our work will allow new efforts in this direction by providing testable hypotheses of what features dolphins might use.

Cetacean vocalisations are highly varied and presumably also of varying function. To analyse these vocalisations and to determine their significance, it is vital to be able to classify them and distinguish calls with biologically distinct origins or functions. Such distinction is necessary to correlate call types with their associated ethological function. This process is unlikely to be possible unless we can identify elements of the signals that contain information relevant to the animals. To develop and test classification methods we need representative data sets of animal vocalizations. In this study, we used a data set balanced for sample size that came from a very specific but artificial context in which dolphins only produce signature whistles. However, in free-swimming dolphins signature whistles only account for around 50% [46]. We now need to conduct further tests on more realistic sets that contain non-signature whistles and unequal sample sizes for each individual to evaluate the usefulness of our method in classifying dolphin signals in the wild. Human visual classification is very successful on such data sets but is time consuming and does not allow easy identification of parameters that contribute to class separation. Automatic techniques such as ARTWARP have been used effectively in recent studies (e.g. [4], [47]) but are still considerably more cumbersome than visual classification, probably because of the high dimensionality of the data being presented to machine learning algorithms. In this study, we used high signal to noise recordings of known individuals to test a new method of whistle classification, which revealed elements of whistles that may have relevance for the way animals perceive them. We hope that our methods could greatly improve our ability to classify these vocalisations, and ultimately decode the information content contained within them.

## References

[1] Caldwell MC, Caldwell DK, Tyack PL. (1990) Review of the signature-whistle hypothesis for the Atlantic bottlenose dolphin. In: Leatherwood S, Reeves RR, The Bottlenose Dolphin. San Diego: Academic Press. 199–234.

[2] Sayigh LS, Esch HC, Wells RS, Janik VM (2007) Facts about signature whistles of bottlenose dolphins, *Tursiops truncatus* . Anim Behav 74: 1631–1642.

[3] Janik VM, Slater PJB (1998) Context-specific use suggests that bottlenose dolphin signature whistles are cohesion calls. Anim Behav 56: 829–838. 9790693 10.1006/anbe.1998.0881

[4] Quick NJ, Janik VM (2012) Bottlenose dolphins exchange signature whistles when meeting at sea. Proc R Soc Lond B Biol Sci 279: 2539–2545. 10.1098/rspb.2011.2537 PMC3350692 22378804

[5] Tyack P (1986) Whistle repertoires of two bottlenosed dolphins, *Tursiops truncatus*: Mimicry of signature whistles? Behav Ecol Sociobiol 18: 251–257.

[6] Harley HE (2008) Whistle discrimination and categorization by the Atlantic bottlenose dolphin (*Tursiops truncatus*): A review of the signature whistle framework and a perceptual test. Behav Processes 77: 243–268. 18178338 10.1016/j.beproc.2007.11.002

[7] Sayigh LS, Tyack PL, Wells RS, Solow AR, Scott MD, et al (1999) Individual recognition in wild bottlenose dolphins: A field test using playback experiments. Anim Behav 57: 41–50. 10053070 10.1006/anbe.1998.0961

[8] Clutton-Brock TH, Albon SD (1979) The roaring of red deer and the evolution of honest advertisement. Behaviour 69: 145–170.

[9] Koren L, Geffen E (2011) Individual identity is communicated through multiple pathways in male rock hyrax (*Procavia capensis*) songs. Behav Ecol Sociobiol 65: 675–684.

[10] Janik VM (1999) Pitfalls in the categorization of behaviour: A comparison of dolphin whistle classification methods. Anim Behav 57: 133–143. 10053080 10.1006/anbe.1998.0923

[11] Henry KS, Gall MD, Bidelman GM, Lucas JR (2011) Songbirds tradeoff auditory frequency resolution and temporal resolution. J Comp Physiol A Neuroethol Sens Neural Behav Physiol 197: 351–359. 21225270 10.1007/s00359-010-0619-0

[12] Zeng FG, Nie K, Stickney GS, Kong YY, Vongphoe M, et al (2005) Speech recognition with amplitude and frequency modulations. Proc Natl Acad Sci USA 102: 2293–2298. 15677723 10.1073/pnas.0406460102 PMC546014

[13] Janik VM, Sayigh L, Wells R (2006) Signature whistle shape conveys identity information to bottlenose dolphins. Proc Natl Acad Sci USA 103: 8293–8297. 16698937 10.1073/pnas.0509918103 PMC1472465

[14] McCowan B, Reiss D (1995) Quantitative comparison of whistle repertoires from captive adult bottlenose dolphins (Delphinidae, *Tursiops truncatus*): A re-evaluation of the signature whistle hypothesis. Ethology 100: 194–209.

[15] Datta S, Sturtivant C (2002) Dolphin whistle classification for determining group identities. Signal Process 82: 251–258.

[16] Nanayakkara SC, Chitre M, Ong S, Taylor E (2007) Automatic classification of whistles produced by indo-pacific humpback dolphins (*Sousa chinensis*). OCEANS 2007-Europe: 1–5.

[17] Buck JR, Tyack PL (1993) A quantitative measure of similarity for *Tursiops truncatus* signature whistles. J Acoust Soc Am 94: 2497–2506. 8270729 10.1121/1.407385

[18] Deecke VB, Janik VM (2006) Automated categorization of bioacoustic signals: Avoiding perceptual pitfalls. J Acoust Soc Am 119: 645–653. 16454318 10.1121/1.2139067

[19] Müllensiefen D, Frieler K (2007) Modelling experts' notions of melodic similarity. Music Sci 11: 183–210.

[20] Lemström K, Wiggins GA. (2009) Formalizing invariances for content-based music retrieval. Proceedings of the 10th International Society for Music Information Retrieval Conference: 591–596.

[21] Parsons D, Levin B. (1975) The directory of tunes and musical themes. Cambridge: S. Brown.

[22] Downie JS (2003) Music information retrieval. Annual review of information science and technology 37: 295–340.

[23] Beyer K, Goldstein J, Ramakrishnan R, Shaft U (1999) When is “nearest neighbor” meaningful? Database Theory—ICDT' 99: 217–235.

[24] Wells RS, Rhinehart HL, Hansen LJ, Sweeney JC, Townsend FI, et al (2004) Bottlenose dolphins as marine ecosystem sentinels: Developing a health monitoring system. EcoHealth 1: 246–254.

[25] D'Errico J. (2012) SLM - shape language modeling. http://www.mathworks.com/matlabcentral/fileexchange/24443-slm-shape-language-modeling (Accessed 2013 Sep 24).

[26] Carpenter GA, Grossberg S (1987) ART 2: Self-organization of stable category recognition codes for analog input patterns. Appl Optics 26: 4919–4930. 10.1364/AO.26.004919 20523470

[27] Zhong S, Ghosh J (2005) Generative model-based document clustering: A comparative study. Knowl Inf Syst 8: 374–384.

[28] Manning CD, Raghavan P, Schütze H. (2008) Introduction to information retrieval. Cambridge: Cambridge University Press.

[29] Everitt B, Landau S, Leese M. (2011) Cluster analysis. Chichester, UK: Wiley.

[30] Krull C, Ranjard L, Landers T, Ismar S, Matthews J, et al (2012) Analyses of sex and individual differences in vocalizations of Australasian gannets using a dynamic time warping algorithm. J Acoust Soc Am 132: 1189. 22894237 10.1121/1.4734237

[31] Ji A, Johnson MT, Walsh EJ, McGee J, Armstrong DL (2013) Discrimination of individual tigers (*Panthera tigris*) from long distance roars. J Acoust Soc Am 133: 1762. 23464045 10.1121/1.4789936

[32] Micó P. (2008) Continuous dynamic time warping. http://www.mathworks.com/matlabcentral/fileexchange/16350-continuous-dynamic-time-warping (Accessed 2013 Sep 24).

[33] Cox TF, Cox MAA. (2000) Multidimensional scaling. Berlin: Springer.

[34] Needleman SB, Wunsch CD (1970) A general method applicable to the search for similarities in the amino acid sequence of two proteins. J Mol Biol 48: 443–453. 5420325 10.1016/0022-2836(70)90057-4

[35] Likic V. (2008) The Needleman-Wunsch algorithm for sequence alignment. Lecture given at the 7th Melbourne Bioinformatics Course, Bi021 Molecular Science and Biotechnology Institute, University of Melbourne.

[36] Ukkonen E (1985) Algorithms for approximate string matching. Information and control 64: 100–118.

[37] Kershenbaum A, Ilany A, Blaustein L, Geffen E (2012) Syntactic structure and geographical dialects in the songs of male rock hyraxes. Proc R Soc Lond B Biol Sci 279: 2974–2981. 10.1098/rspb.2012.0322 PMC3385477 22513862

[38] Pauws S (2002) Cuby hum: A fully operational query-by-humming system. ISMIR 2002 Conference Proceedings, IRCAM 2002: 187–196.

[39] Müllensiefen D, Frieler K (2004) Cognitive adequacy in the measurement of melodic similarity: Algorithmic vs. human judgments. Computing in Musicology 13: 147–176.

[40] Garrett A. (2003) Fuzzy ART and fuzzy ARTMAP neural networks. http://www.mathworks.com/matlabcentral/fileexchange/4306-fuzzy-art-and-fuzzy-artmap-neural-networks(Accessed 2013 Sep 24).

[41] Thornton A, Clayton NS, Grodzinski U (2012) Animal minds: From computation to evolution. Philos Trans R Soc Lond B Biol Sci 367: 2670–2676. 22927565 10.1098/rstb.2012.0270 PMC3427558

[42] Fripp D, Owen C, Quintana-Rizzo E, Shapiro A, Buckstaff K, et al (2005) Bottlenose dolphin (*Tursiops truncatus*) calves appear to model their signature whistles on the signature whistles of community members. Anim Cogn 8: 17–26. 15221637 10.1007/s10071-004-0225-z

[43] Charlton BD, Zhihe Z, Snyder RJ (2009) Vocal cues to identity and relatedness in giant pandas (*Ailuropoda melanoleuca*). J Acoust Soc Am 126: 2721–2732. 19894848 10.1121/1.3224720

[44] Boughman J, Moss C. (2003) Social sounds: Vocal learning and development of mammal and bird calls. In: Simmons AM, Fay RR, Popper AN, Acoustic Communication. New York: Springer. 138–224.

[45] Ralston JV, Herman LM (1995) Perception and generalization of frequency contours by a bottlenose dolphin (*Tursiops truncatus*). J Comp Psychol 109: 268–277.

[46] Cook ML, Sayigh LS, Blum JE, Wells RS (2004) Signature-whistle production in undisturbed free-ranging bottlenose dolphins (*Tursiops truncatus*). Proc R Soc Lond B Biol Sci 271: 1043–1050. 10.1098/rspb.2003.2610 PMC1691697 15293858

[47] Deecke VB, Nykänen M, Foote AD, Janik VM (2011) Vocal behaviour and feeding ecology of killer whales *Orcinus orca* around Shetland, UK. Aquatic Biology 13: 79–88.

